# Preservation of the Hypoxic Transcriptome in Glioblastoma Patient-Derived Cell Lines Maintained at Lowered Oxygen Tension

**DOI:** 10.3390/cancers14194852

**Published:** 2022-10-04

**Authors:** Agata Gozdz, Bartosz Wojtaś, Patrycja Szpak, Paulina Szadkowska, Tomasz Czernicki, Andrzej Marchel, Katarzyna Wójtowicz, Wojciech Kaspera, Piotr Ladzinski, Wojciech Szopa, Marcin Niedbala, Sergiusz Nawrocki, Bozena Kaminska, Ilona Kalaszczynska

**Affiliations:** 1 Department of Histology and Embryology, Centre for Biostructure Research, Medical University of Warsaw, 02-004 Warsaw, Poland; 2 Laboratory of Molecular Neurobiology, Nencki Institute of Experimental Biology, Polish Academy of Sciences, 02-093 Warsaw, Poland; 3 Laboratory of Sequencing, Nencki Institute of Experimental Biology, Polish Academy of Sciences, 02-093 Warsaw, Poland; 4 Postgraduate School of Molecular Medicine, Medical University of Warsaw, 02-091 Warsaw, Poland; 5 Department of Neurosurgery, Medical University of Warsaw, 02-097 Warsaw, Poland; 6 Department of Neurosurgery, Central Teaching Clinical Hospital, 02-097 Warsaw, Poland; 7 Department of Neurosurgery, Medical University of Silesia, Regional Hospital, 04-730 Sosnowiec, Poland; 8 Department of Oncology, Collegium Medicum, University of Warmia and Mazury in Olsztyn, 10-228 Olsztyn, Poland; 9 Laboratory for Cell Research and Application, Medical University of Warsaw, 02-097 Warsaw, Poland

**Keywords:** glioblastoma, hypoxia, 5% oxygen, glioblastoma stem cells, cell cycle, DNA repair, inflammation, temozolomide, spheroid, RNA sequencing

## Abstract

**Simple Summary:**

The extent of tumour oxygenation is crucial for glioblastoma progression and the effectiveness of radio- and chemotherapy. Patient-derived in vitro cell cultures are the mainstay of molecular biology research, the discovery of new therapeutic targets, and drug testing. Therefore, mirroring as many aspects of in vivo settings as possible, including oxygen concentration, is desired. Here we analyse the effect of oxygen tension on the transcriptome of numerous patient-derived GBM cells and demonstrate that cells cultured in lowered oxygen tension express more genes indicative of higher levels of hypoxia, metabolic adaptation, stemness and tumour progression than cells growing in standard, atmospheric oxygen concentration. The same transcriptomic pattern was also found in primary GBM samples. Its specificity for GBMs was confirmed using the public TCGA dataset. Our data strongly argue for the benefit of lower oxygen tension during culturing of patient-derived GBM cells to preserve oxygen-sensitive pathways in GBM. The proposed approach better mimics certain aspects of GBM pathophysiology than traditional cultures and may advance GBM research in finding a cure.

**Abstract:**

Despite numerous efforts aiming to characterise glioblastoma pathology (GBM) and discover new therapeutic strategies, GBM remains one of the most challenging tumours to treat. Here we propose the optimisation of in vitro culturing of GBM patient-derived cells, namely the establishment of GBM-derived cultures and their maintenance at oxygen tension mimicking oxygenation conditions occurring within the tumour. To globally analyse cell states, we performed the transcriptome analysis of GBM patient-derived cells kept as spheroids in serum-free conditions at the reduced oxygen tension (5% O_2_), cells cultured at atmospheric oxygen (20% O_2_), and parental tumour. Immune cells present in the tumour were depleted, resulting in the decreased expression of the immune system and inflammation-related genes. The expression of genes promoting cell proliferation and DNA repair was higher in GBM cell cultures when compared to the relevant tumour sample. However, lowering oxygen tension to 5% did not affect the proliferation rate and expression of cell cycle and DNA repair genes in GBM cell cultures. Culturing GBM cells at 5% oxygen was sufficient to increase the expression of specific stemness markers, particularly the PROM1 gene, without affecting neural cell differentiation markers. GBM spheroids cultured at 5% oxygen expressed higher levels of hypoxia-inducible genes, including those encoding glycolytic enzymes and pro-angiogenic factors. The genes up-regulated in cells cultured at 5% oxygen had higher expression in parental GBMs compared to that observed in 20% cell cultures, suggesting the preservation of the hypoxic component of GBM transcriptome at 5% oxygen and its loss in standard culture conditions. Evaluation of expression of those genes in The Cancer Genome Atlas dataset comprising samples of normal brain tissue, lower-grade gliomas and GBMs indicated the expression pattern of the indicated genes was specific for GBM. Moreover, GBM cells cultured at 5% oxygen were more resistant to temozolomide, the chemotherapeutic used in GBM therapy. The presented comparison of GBM cultures maintained at high and low oxygen tension together with analysis of tumour transcriptome indicates that lowering oxygen tension during cell culture may more allegedly reproduce tumour cell behaviour within GBM than standard culture conditions (e.g., atmospheric oxygen tension). Low oxygen culture conditions should be considered as a more appropriate model for further studies on glioblastoma pathology and therapy.

## 1. Introduction

Glioblastoma (GBM, World Health Organization IV grade *IDH*-wild type glioma) is one of the deadliest human malignancies in adults [1]. Despite the aggressive treatment comprised of tumour resection, irradiation and chemotherapy, the overall survival of individuals diagnosed with primary glioblastoma is 12-18 months. Less than 10% of patients survive beyond five years [2]. The currently used therapeutic regimen developed and described by Stupp et al. in 2005 is the most effective so far for primary GBMs [3], with some modifications regarding temozolomide dosing [1]. Despite the immense scientific and clinical efforts, there is no effective GBM treatment [1,2].

Heterogeneity and plasticity of glioblastoma at the genetic, epigenetic, transcription and translation levels are among the main impediments to GBM eradication [4,5,6,7,8]. The phenomena mentioned above are deeply affected by varied and dynamically changing oxygen partial pressure within the tumour [9,10]. GBM progression and response to radio- and chemotherapy are strongly influenced by tumour oxygenation, with intratumoral hypoxia being the negative predictor of the effectiveness of GBM treatment and patient survival [11,12]. Angiogenesis inhibitors targeting hypoxia-dependent Vascular Endothelial Growth Factor (VEGF) action are currently used for recurrent GBMs [13]. Novel techniques for precise detection of hypoxic regions and compounds able to increase intratumoural oxygen levels or counteract molecular changes induced by hypoxia are currently evaluated in pre-clinical GBM models and clinical trials in GBM patients [10,14,15,16,17,18].

Physiological intra-tissue oxygen pressure within a healthy human brain ranges from 25 to 48 mm Hg, values corresponding to 2–8% interstitial oxygen, thus much lower than measured in ambient air (160 mm Hg) and lung alveoli (110 mm Hg) [19]. The oxygen measurements in glioma tissue during surgery revealed variable levels of GBM oxygenation, ranging from values typical for healthy brain tissue (up to 10% oxygen) to moderately hypoxic (0.5% oxygen), severely hypoxic (0.1% oxygen) and anoxic [11]. This “oxygenation heterogeneity” was later confirmed by studies using chemical oxygen probes and the detection of transcripts or proteins induced by hypoxia at the particular tumour areas (e.g., *CA9*, *VEGF*, *ANGPT2*, *EPAS1*), with the rest of the tumour being relatively well-oxygenated [20,21,22]. Oxygen concentration in standard 5% CO2 incubator reaches nearly 20% [19]. The vast majority of in vitro GBM cellular models are maintained in later conditions. Importantly, culturing human cells, including U87 MG glioma cells, at atmospheric oxygen may elicit unphysiological responses. Antioxidant defence, DNA repair, and mitochondrial function are grossly affected by the excess of oxygen [23,24], strongly arguing for maintaining oxygen partial pressure in in vitro cell cultures closer to that observed in tissues. 

The current study aimed to test whether GBM-derived cells cultured in reduced oxygen conditions (5% oxygen) retain the transcriptomic patterns from original tumours better than those maintained under standard (atmospheric oxygen) conditions. GBM cells experiencing reduced oxygen conditions produced more stem cell markers, such as *PROM1* and *CD44* [25,26,27,28,29]. Here we confirmed the effect of oxygen levels on *PROM1* expression. To obtain further insight into oxygen-dependent gene expression, we analysed the global transcriptome of freshly resected GBMs and corresponding GBM-derived cultures maintained at 5% or 20% oxygen. The analysis showed strong enrichment in cell cycle and DNA repair pathways in GBM cells compared to the parental tumour. There was no substantial difference in cell cycle and no global alterations to DNA replication and DNA repair genes expression depending on oxygenation conditions, complemented by the lack of differences in cell cycle distribution. GBM cells cultured at 5% oxygen express higher levels of hypoxia-inducible genes, like those for glycolytic enzymes and pro-angiogenic factors. The expression pattern for those genes in tumour tissue and GBM cells cultured at 5% oxygen was similar and differed from that observed in cells cultured in standard conditions, e.g., 20% oxygen. Moreover, GBM cells cultured at 5% oxygen were more resistant to temozolomide. Patient-derived cell cultures kept under low oxygen tension should be considered a more relevant in vitro model for glioblastoma pathobiology and drug testing studies.

## 2. Materials and Methods

### 2.1. Human Subjects

GBM specimens were collected from patients undergoing surgery at the Medical University of Warsaw Neurosurgery Clinic and at the Regional Hospital of the Medical University of Silesia in Sosnowiec. The study was approved by the Bioethics Committee of the Medical University of Silesia (permit number KNW/0022/KB1/2/I/17). All patients participating in the study provided informed consent prior to the surgery. Histological examination classified specimens as glioblastoma. Patient information is provided in Table 1.

### 2.2. Materials

DMEM F12 Glutamax Supplement, B27 Supplement, human epidermal growth factor (EGF), human basic fibroblast growth factor (bFGF), collagenase IV, Presto Blue Cell Viability Reagent, foetal bovine serum (FBS) and 4′,6-Diamidino-2-phenylindole dihydrochloride (DAPI) were from Thermo Fisher Scientific (Waltham, MA, USA). DNAse, laminin, Penicillin–Streptomycin solution, and ACCUMAX were ordered from Sigma–Aldrich (Saint Louis, MO, USA). Temozolomide was purchased from Sigma-Aldrich and dissolved in DMSO. ZAPR-100 Red Blood Cell Lysing Buffer was from INCELL Corporation LLC (San Antonio, TX, USA), and heparin was from STEMCELL Technologies Inc. (Vancouver, BC, Canada).

### 2.3. GBM Tissue Processing, GBM Cell Cultures Establishment and Maintenance

GBM tissue was processed one to six hours after resection. Some portion of the tissue was quick frozen for later nucleic acids isolation, and the rest was digested with collagenase IV and DNAse (adopted from [30]). The culture medium was composed of DMEM-F12 with Glutamax, B27 supplement, 20 ng/mL EGF, 20 ng/mL bFGF, Penicilin-Streptomycin solution, and 1 IU/mL heparin. Cells were seeded onto cell culture flasks dedicated to suspension cultures or flasks coated with laminin (0.5 ug per cm2) to achieve adherent culture conditions. Immediately after displaying an exponential growth rate, cells were propagated for cryopreservation and the day of the first cryopreservation was acknowledged as the date of cell line establishment. GBM cells were cultured since derivation in the standard culture conditions, e.g., in the incubator at atmospheric (20%) O_2_, 5% CO_2_, 37 °C or in the BioSpherix Xvivo System (Parish, NY, USA) at 5% O_2_, 5% CO_2_, 37 °C.

### 2.4. Cell Treatment and Viability Assay

Cells were seeded at the density of 5000 cells per well of 96 well culture plates coated with laminin to obtain adherent conditions or uncoated plates for cells growing as spheroids. Two days after seeding, both spheroid and adherent cells were treated with temozolomide or a relevant amount of solvent (DMSO). Presto Blue Cell Viability Reagent was added after 72 h, and the amount of metabolised reagent was determined by OD measurement at 535 nm. All experiments were performed two or three times in triplicates, and the results were expressed as a percentage of control, e.g., the value measured in cells incubated with DMSO. A two-way ANOVA test with Sidak’s correction was performed using GraphPad Prism v9.3.1 for Windows (GraphPad Software, San Diego, CA, USA).

### 2.5. Flow Cytometry

Cells growing as spheres were enzymatically dissociated and fixed in cold 80% ethanol. Next, they were washed in cold PBS with 0.5% FBS, and stained for 30′ with 10 μg/mL DAPI. The intensity of DAPI staining per cell was determined with a CytoFLEX flow cytometer (Beckman Coulter Inc., Indianapolis, IN, USA) using a PB450 laser. Cell cycle distribution was evaluated with FlowJo v10 for Windows (BD Life Sciences, Franklin Lakes, NJ, USA).

### 2.6. Nucleic Acids Isolation

Cells cultured at 20% and 5% oxygen were harvested at the same or adjacent passage, mechanically disrupted in TRIzol Reagent (Thermo Fisher Scientific), and processed along the manufacturer’s instructions to obtain material for RNA sequencing.

### 2.7. RNA Sequencing and Analysis

The quality and quantity of isolated nucleic acids were determined by Nanodrop (Thermo Fisher Scientific, Waltham, MA, USA) and Agilent Bioanalyzer (Agilent Technologies, Santa Clara, CA, USA). mRNA libraries were prepared using KAPA Stranded mRNAseq Kit (Roche, Basel, Switzerland) according to the manufacturer’s protocol (KR0960-v6.17). mRNAs were enriched from 500 ng of total RNA using poly-T oligo-attached magnetic beads (Kapa Biosystems, MA, USA). Enriched mRNA was fragmented, and then the first and second strands of cDNA were synthesised. Consequentially adapters were ligated and the loop structure of each adapter was cut by the USER enzyme (NEB, Ipswich, MA, USA). Finally, the amplification of obtained dsDNA fragments that contained specific adapter sequences was performed using NEB starters (Ipswich, MA, USA). Quality control of final libraries was performed using Agilent Bioanalyzer High Sensitivity dsDNA Kit (Agilent Technologies, Palo Alto, CA, USA). Concentrations of the final libraries were measured using Quantus Fluorometer and QuantiFluor ONE Double-Stranded DNA System (Promega, Madison, WI, USA). Libraries were sequenced on HiSeq 1500 (Illumina, San Diego, CA, USA) on the rapid run flow cell with paired-end settings (2x76bp).

Data analysis: RNA sequencing reads were aligned to the human genome with the STAR algorithm (https://www.ncbi.nlm.nih.gov/pmc/articles/PMC3530905/, accessed on 5 February 2020), a fast gap-aware mapper. Then, gene counts were obtained by feature counts (10.1093/nar/gkz114) using human genome annotations. The counts were imported to R and processed by DESeq2 [31]. The counts were normalised for gene length, and library size and statistical analysis was performed for the following comparisons: (1) 5% oxygen tumour-derived cell lines vs. tumour; (2) 20% oxygen tumour-derived cell lines vs. tumour; (3) 5% oxygen tumour-derived cell lines vs. 20% oxygen tumour-derived cell lines. KEGG Gene pathways analyses were performed using clusterProfiler R library [32]. 

TCGA public data analysis: TCGA level 3 RNAseq data (aligned by STAR and gene expression counted by HTseq) were uploaded to R. Data from TCGA GBM (glioblastoma, WHO grade IV) and LGG (lower-grade gliomas, WHO grade II/III) repositories were uploaded. Gene expression levels as FPKM (fragments per kilobase of exon per million) were used for further analysis. Clinical data for LGG and GBM datasets were obtained from the work of Ceccarelli et al. 2016 (Table S1 within referenced article) [33]. Statistical analysis and visualisation were performed in R. The curated sets of genes characteristic for each GBM subtype, categorised originally by Verhaak et al. [34], were downloaded from the Molecular Signatures Database v7.5.1. The analysis for the following gene sets was performed: VERHAAK_GLIOBLASTOMA_PRONEURAL, VERHAAK_GLIOBLASTOMA_NEURAL, VERHAAK_GLIOBLASTOMA_CLASSICAL, VERHAAK_GLIOBLASTOMA_MESENCHYMAL, HALLMARK_EPITHELIAL_MESENCHYMAL_TRANSITION. 

## 3. Results

**Figure 1:**
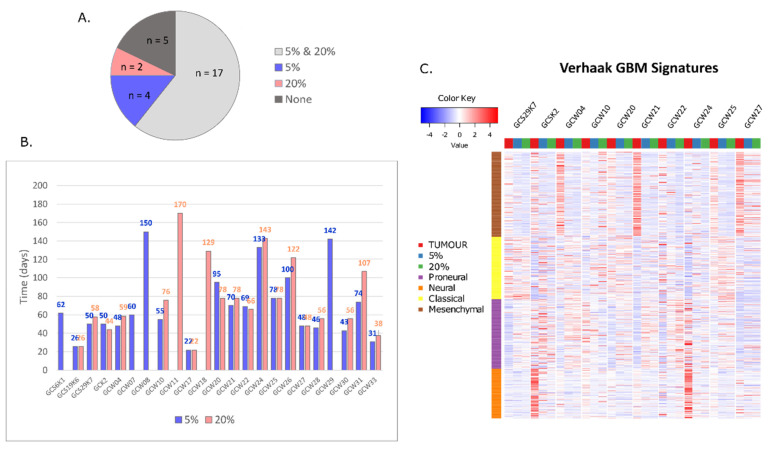
**Efficiency and time required for developing GBM-derived cell cultures**. Cell cultures derived from 28 freshly resected GBM fragments were maintained at 5% and 20% oxygen. (**A**) Graph depicts a number of cultures obtained at a single oxygen condition (5% or 20% oxygen), both conditions (5% and 20% oxygen) and a number of failed attempts. (**B**) The time required for cell line establishment is calculated in days, from a day of tumour isolation till the first biobanking (details in Methods and Materials section). Twenty cultures were grown as spheroids, and four cultures (SOS19K6, WUM17, WUM18 and WUM33) were grown as adherent cells. (**C**) Heatmap depicting genes characteristic for four GBM subtypes in 10 GBMs, 5% oxygen culture and 20% oxygen culture.

The applied cell culture conditions allow the preservation and propagation of “glioma stem-like cells”(GSC) [35,36,37]. Out of 28 glioblastoma specimens, we developed cultures from 23 tumours. In the case of 17 GBM specimens, the same cell cultures were kept at 5% and 20% oxygen; for two GBM specimens, the cell culture was developed only at 20% oxygen, and for four GBMs only at 5% oxygen (Figure 1A). Cells growing as spheres usually require a shorter time to generate a cell line when maintained at 5% oxygen (Figure 1B). The attempt to establish cell cultures at 1% oxygen had failed as cells died after a few weeks in vitro. The information on cell culturing conditions (spheroid or adherent) is provided in Table 1. GBM specimens from 10 patients and relevant cell cultures analysed by sequencing, are highlighted in blue (Table 1).

**Figure 2:**
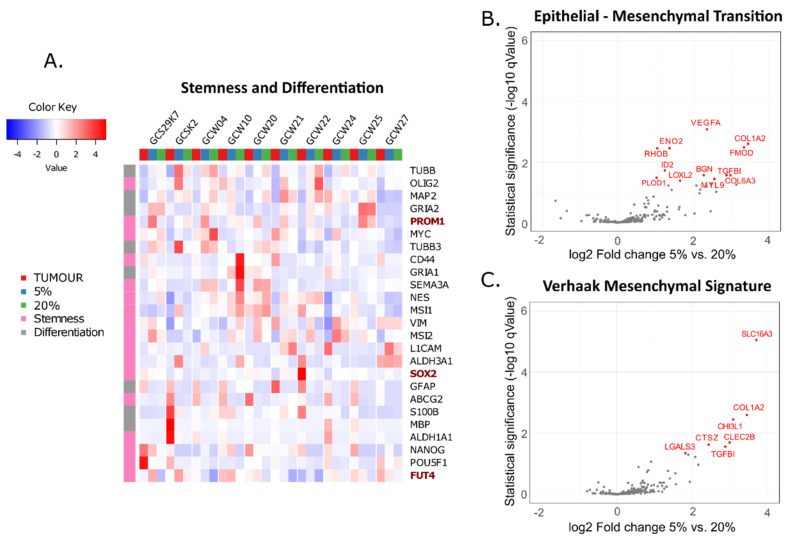
**Expression of stemness and differentiation marker and EMT genes in GBMs and cells cultured at 5% and 20% oxygen.** (**A**) Heatmap shows an expression of selected stemness (pink) and differentiation (grey) genes in tumour and its derivative cell lines at 5% oxygen and 20% oxygen, with row clustering applied. (**B**,**C**) Volcano plots depict the expression of a group of EMT genes and mesenchymal GBM subtype genes in cells grown at 5% and 20% oxygen. Genes differentially expressed between conditions (expression higher than log2 Fold equal to 0.5, and adjusted p lower than 0.05) are annotated in red.

Culturing cells as spheres allows for the enrichment of GSCs. Studies on GSCs demonstrated that these cells are more resistant to anti-glioblastoma therapies than adherent glioma cells contributing to GBM relapse after initial treatment [38,39,40]. The expression of *PROM1*, a stem cell marker, was higher in GBM cells cultured at 3% to 7% oxygen [25,26,27,41]. We determined the expression of selected stemness and differentiation markers in cells cultured at 5% and 20% oxygen. Out of the panel of 25 genes, only *PROM1*, *FUT4*, and *SOX2* showed higher expression at 5% oxygen (Figure 2A). None of the markers of differentiated neural cells has changed their expression significantly in an oxygen level-dependent manner (Figure 2A). Epithelial-Mesenchymal Transition (EMT) is an important phenomenon fuelling GBM progression and worsening patient outcomes. It comprises reversible, epigenetic and phenotypic alterations leading to increased cell migration, tumour invasion, and therapy resistance [42]. Low oxygen was shown to be among EMT- inducing stimuli in glioblastoma cells in vitro and in vivo [43,44]. Therefore, we checked whether culturing GBM cells at 5% affects the expression of genes grouped in the curated EMT gene set (HALLMARK_EPITHELIAL_MESENCHYMAL_TRANSITION), consisting of transcription factors, cytokines, proteases, extracellular matrix components etc. The expression of several genes belonging to the EMT set increased significantly (Figure 2B). Along with the classification proposed by Verhaak et al., the portion of GBM tumours presents with enhanced mesenchymal markers expression and falls into the “mesenchymal subtype” [34]. We employed the list of genes characteristic of mesenchymal GBM subtype to compare the expression of these genes in 5% and 20% GBM cell lines and found that five additional genes displayed higher expression levels in cells cultivated at 5% oxygen (Figure 2C). These data indicate that lowering oxygen tension to 5% in spheroid GBM culture enhances the expression of EMT-related genes, which is in line with some previous reports on EMT and oxygenation levels in GBM.

**Figure 3:**
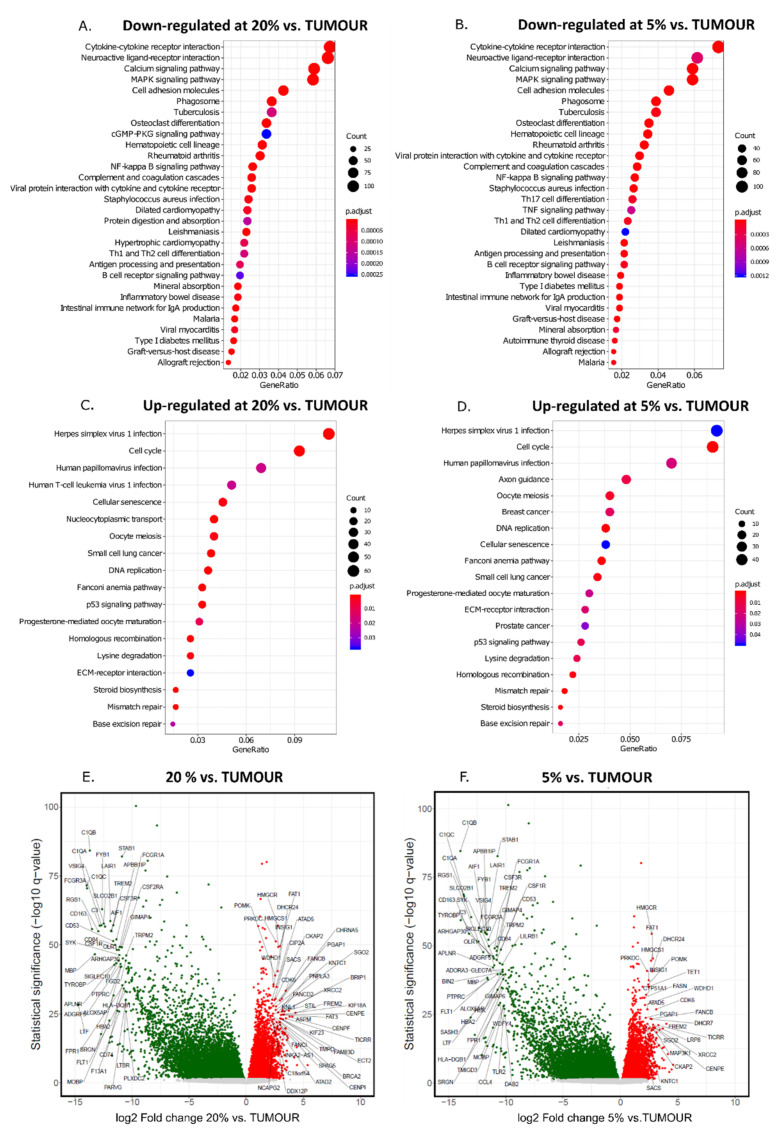
**Most molecular pathways and genes differentiating transcriptomic profiles in cell cultures and corresponding GBMs.** KEGG pathways enrichment analysis of genes differentially expressed in culture and a corresponding tumour. (**A**) Genes most down-regulated in 20% cell cultures vs. tumours. (**B**) Genes most down-regulated in 5% cell cultures vs. tumours. (**C**) Genes most up-regulated in 20% cell cultures vs. tumour. (**D**) Genes most up-regulated in 5% cell cultures vs. tumours. Black circle diameter reflects a number of up- or down-regulated genes belonging to the particular KEGG pathway. Volcano plots showing the global difference in gene expression between 20% culture and tumour (**E**) and 5% culture and tumour (**F**). Genes with the lowest and highest difference in the particular cell lines set and tumour are annotated on the plots, e.g., genes for which log2 fold change was below −10.5 and above 2.25, adjusted *p*-value < 1 × 10^−20^ for 20% oxygen cultures vs. tumour, and genes for which log2 fold change was below -10 and above 2 and adjusted *p*-value < 1 × 10^−20^ for 5% oxygen cultures vs. tumour).

GBM is a highly complex tumour composed of rare GSCs, more differentiated tumour cells, infiltrating microglia and peripheral immune cells, and endothelial cells [45,46]. We performed RNA expression analysis of parental tumour and corresponding cell cultures established at 5% and 20% oxygen to determine global transcriptomic profiles and analyse functional responses. KEGG pathways enrichment analysis demonstrates that inflammatory response, antigen presentation, and synaptic transmission genes are most downregulated in cell cultures, compared to the original GBM sample, and it occurs both at 5% and 20% oxygen (Figure 3A,B and Supplementary Table S1). The expression of the cell cycle, DNA replication and specific systems of DNA repair genes and steroid biosynthesis pathway genes was increased in cell cultures regardless of oxygen levels (Figure 3C,D, Supplementary Table S1). These data highlight both crucial features of GBM, e.g., accumulation and activation of immune cells and variable states of glioblastoma cell differentiation within the tumour as well as properties of cultured cells, e.g., growth advantage of clones expressing high levels of genes enabling cell cycle progression and DNA repair.

The volcano plots comparing the distribution of gene expression in GBM and cell cultures are highly asymmetrical, implying the transcriptional complexity of the tumour sample and relative simplification of gene expression pattern in cells cultured under defined conditions (Figure 3E,F). Featured genes on the far left side of both plots represent mainly effectors and regulators of immune cell function, and genes coding for proteins enabling cell cycle progression and DNA repair predominate on the far right portion of the plot (Figure 3E,F). 

**Figure 4:**
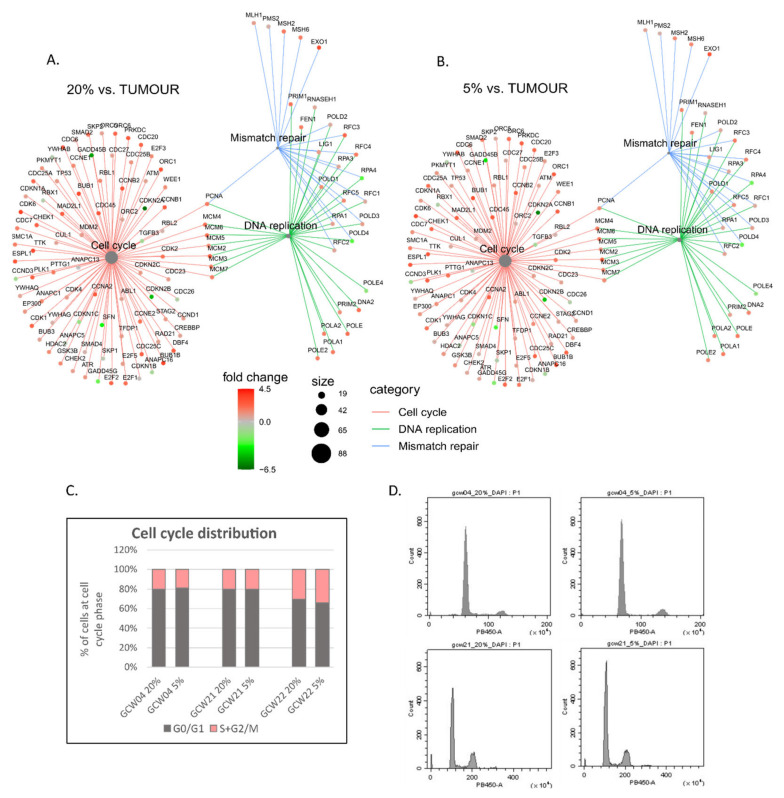
**Expression of cell cycle and DNA repair genes in GBM cell cultures and parental tumours.** Cnetplots of selected KEGG pathways genes comparing gene expression in GBMs and 20% (**A**) or 5% (**B**) cell cultures. Black dots represent a number of statistically differentially expressed genes in the given pathway in the particular comparison. (**C**) Flow cytometry analysis of DNA content in GCW04, GCW21 and GCW22 cell cultures maintained at 5% or 20% oxygen. Cell cycle distribution is shown as a percentage of cells at the indicated cell cycle phase. (**D**) Exemplary histograms showing DNA content in three cell cultures kept at 20% or 5% oxygen.

There is no consensus regarding the effect of hypoxia on GBM cell proliferation, largely due to the diversity of employed experimental models. It was shown that mild hypoxia (1% oxygen) limits the proliferation of cultured GBM cells [47], increases proliferation [48,49] or has no effect [50]. Detailed analysis of the KEGG pathway comprising cell cycle and DNA replication genes showed that most of the genes are equally up-regulated at 5% and 20% oxygen cultures compared to GBM (Figure 4A,B). However, several genes were strongly downregulated, including *CDKN2A* and *CDKN2B*, which encode potent inhibitors of cyclin-dependent kinases. Genes related to DNA repair systems, e.g., Fanconi Anaemia system, homologous recombination system and mismatch repair system had higher expression in 5% and 20% GBM cell cultures than in corresponding tumours (Figure 4A,B). The analysis of expression of cell cycle genes in three GBM-derived cells cultured at 5% and 20% showed no substantial effect of oxygen level on cell cycle distribution (Figure 4C,D).

**Figure 5:**
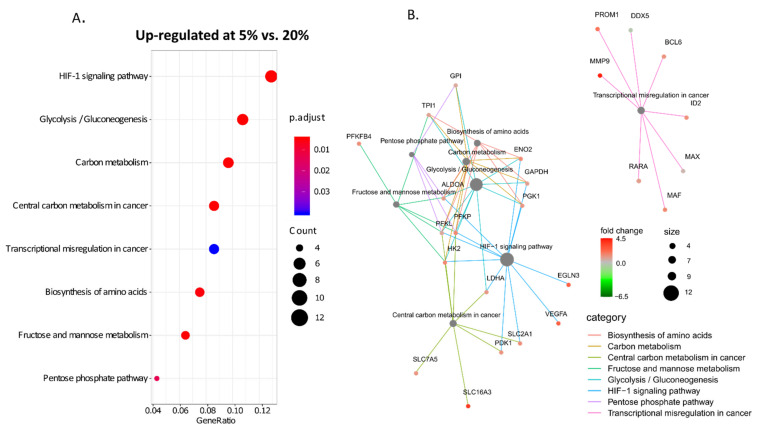
**Most distinct molecular pathways in cell cultures maintained at 5% and 20% oxygen.** (**A**) KEGG pathways most up-regulated in 5% vs. 20% cell cultures. (**B**) Cnetplot showing genes from most differentiating KEGG pathways. Black dots represent a number of genes falling into the pathway category from those statistically differentially expressed between 5% and 20% cell cultures.

Next, KEGG pathways enrichment analysis was performed for cell cultures only. We found several pathways up-regulated in cultures kept at 5% oxygen (Figure 5A, Supplementary Table S2), but no particular KEGG pathway in which genes were downregulated. The pathways up-regulated at 5% oxygen were those related to hypoxia-inducible factor (HIF)-1 signalling and glucose and carbon metabolism (Figure 5A). The exemplary genes from HIF-1- related metabolic pathways are shown in Figure 5B. Moreover, several genes belonging to the category “Transcriptional misregulation in cancer” coding for proteins important for cancer progression had higher expression at 5% oxygen, including *MMP9*, *BCL6*, *RARA*, *MAF*, *ID2* and *PROM1* (Figure 5B). The genes with expression levels different at least two-fold in 5%, and 20% O_2_ cultures and having a *p*-value for this difference lower than 1 × 10^−5^ are listed in Supplementary Table S3.

**Figure 6:**
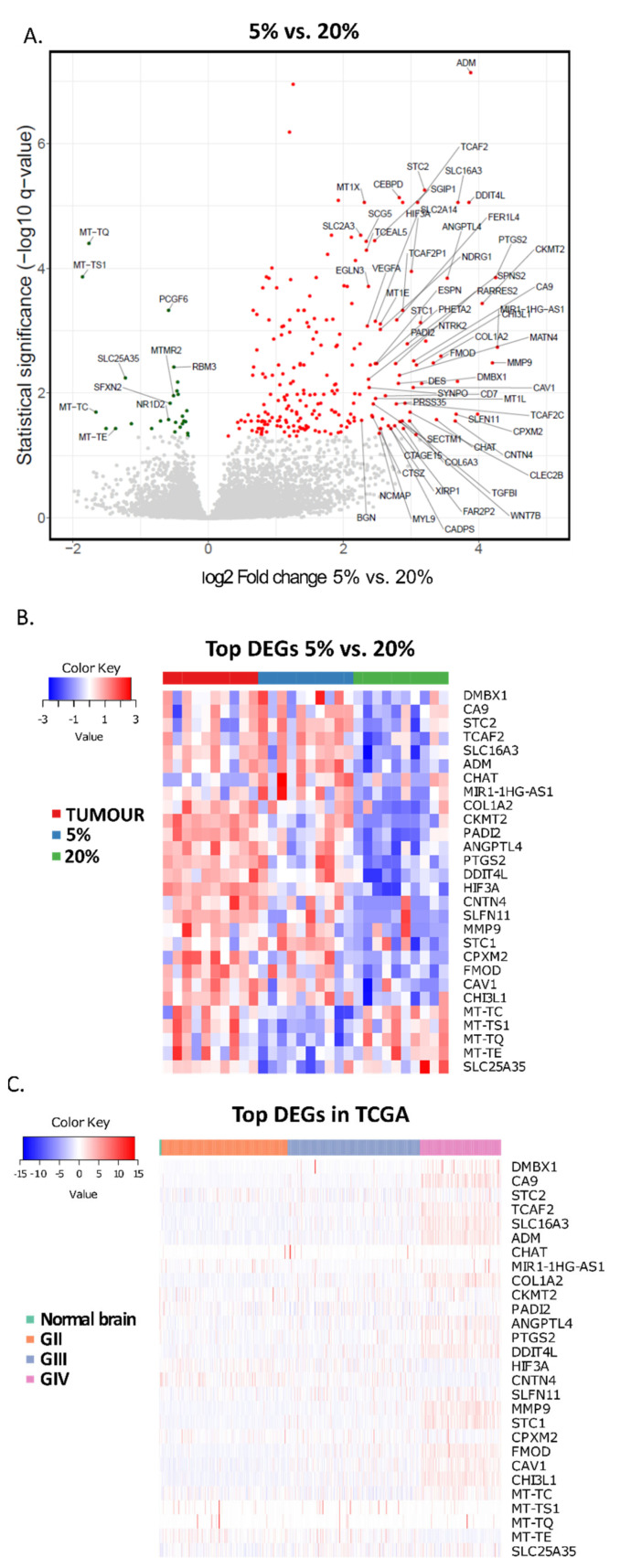
Identification of 30 most differentially expressed genes (DEGs) in tumours, cell cultures, and the TCGA dataset. (**A**) Volcano plots showing a global difference in gene expression between 20% culture and 5% culture. Genes with the lowest and highest expression. Cut-off just for the colour of significance is adjusted *p*-value < 0.05. For the genes annotated on the plot log2 fold change was below-0.5 and above 2.25 for genes down-regulated and up-regulated in 5% oxygen cultures, respectively. (**B**) Heatmap created for 30 most differentially expressed genes in the tumour, 5% and 20% cell cultures. (**C**) Heatmap depicting expression levels of top 30 DEGs in the TCGA dataset comprising normal brain, and gliomas of WHO grades I–IV.

Comparing global gene expression in 5% and 20% O_2_ GBM cultures revealed higher proportions of strongly up-regulated genes in lowered oxygen tension (Figure 6A). Only a few genes were significantly downregulated, mostly belonging to mitochondrial transcriptome and encoding mitochondria-specific tRNA molecules. The list of 30 differentially expressed genes (DEGs) is presented in Figure 5A. The group of genes with the highest score at 5% oxygen comprises those encoding angiogenesis/vasculogenesis regulators (*ANGPTL4*, *ADM*), immunomodulators (*CHI3L*, *PTGS2*), extracellular space acidifying enzyme (*CA9*), secreted proteins regulating calcium/phosphate homeostasis (*STC1*, *STC2*), ECM-related and cell-cell adhesion molecules (*COL1A2*, *CNTN4*, *FMOD*), transcription regulators (*DMBX1*, *HIF3A*), caveolae associated protein (*CAV1*) (Figure 6B). Profiles of the top 30 DEGs between 5% and 20% expression in ten GBM samples and established 5% and 20% O_2_ cell cultures demonstrated that most up-regulated genes clustered together in 5% O_2_ cultures and tumours (Figure 6B).

Analysis of this set of genes in a large number of glioma TCGA samples revealed that the expression of most genes from the top 30 group is higher in GBMs than their expression in lower-grade gliomas and normal brains (Figure 6C). The genes with the most significantly reduced expression in 5% cultures are mitochondria-associated and mitochondrial DNA coded genes (Figure 6A,B). However, these are different genes from the most GBM differentially regulated in the TCGA dataset (Figure 6B,C).

**Figure 7:**
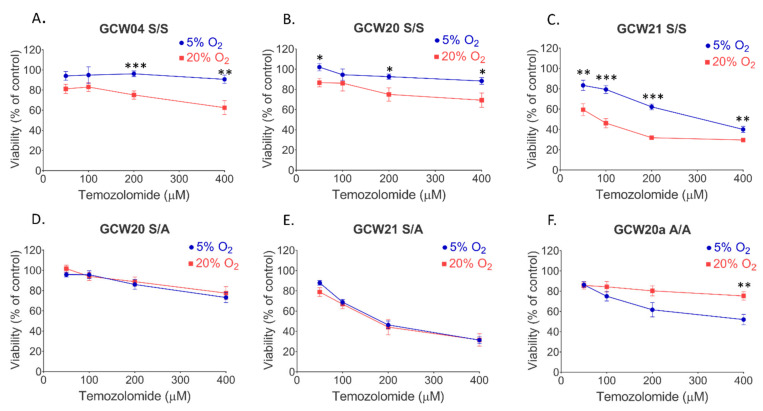
**Effects of oxygen tension on temozolomide toxicity.** GBM cells cultured at 5% or 20% oxygen were treated for 72 hr with TMZ at increasing concentrations or a relevant amount of DMSO as a solvent. Cell viability was calculated as a percentage of the viability of vehicle-treated cultures. (**A**–**C**) GBM cell cultures s GCW04, GCW20 and GCW21 maintained at 5% or 20% oxygen were seeded and treated as spheres. (**D**,**E**) GCW20 and GCW21 cell lines were seeded and treated as adherent cultures. (**F**) GCW20a cells were cultured from the beginning as a monolayer. All experiments were repeated two or three times. Data are presented as mean, error bars represent S.E. Statistical significance was evaluated with 2-way ANOVA with Sidak’s correction, * *p* < 0.05, ** *p* < 0.01, *** *p* < 0.001.

Hypoxia may reduce the sensitivity of GBM to radiotherapy and chemotherapy [10,14]. Therefore, we explored how GBM spheres cultured at 5% oxygen respond to temozolomide (TMZ). Three cell cultures growing at 5% or 20% oxygen were challenged with increasing concentrations of TMZ. Cell metabolic activity was determined 72 h later. Lowering oxygen tension diminished TMZ toxicity in all 3 GBM cultures (Figure 7A–C). RNA-seq results may partially explain this reduced sensitivity of cells grown at 5% oxygen, as mRNA level for *MGMT*, the gene coding for O^6^-methylguanine DNA methyltransferase, was 2.3 times higher in cells grown at 5% oxygen. However, the difference was not significant due to the high variation between cell cultures (not shown).

Nevertheless, the exact mechanism of oxygen-dependent response to TMZ remains to be clarified. Interestingly, the protective effect of low oxygen was restricted to cells grown as spheroids. It is worth noting that the protective effect of lower oxygen was lost when cells were seeded as adherent cells (Figure 7D–F). This result points to the importance of spatial interactions between cultured cells, combined with reduced oxygen tension and hypoxic gradient in 3D systems.

## 4. Discussion

Here we characterise the transcriptome of GBM patient-derived cell cultures, which were established and maintained at lowered oxygen tension to resolve their suitability as an alternative to traditional in vitro models for research on glioblastoma pathobiology. Since the proposed transcription-based subtyping of GBMs [34,51], some attempts to reproduce a subtype of the parental tumour in in vitro culture were made, with only partial success [52,53]. The results of the current study point to the same conclusion. Recent reports underscored the importance of microenvironmental clues for sustaining the mesenchymal subtype of experimental GBM and the significant contribution of invading immune cells to mesenchymal subtype characteristics [54,55]. The comparison of the global transcriptome of parental tumour and derivative cell lines shows a reduced expression of immune/inflammatory response genes and markers of differentiated neural cells in GBM-derived cell cultures (Figure 3). This profound divergence in gene expression patterns may be explained by the depletion of immune cells during the establishment and propagation of GBM cultures. Furthermore, cell cultures are rapidly dominated by the clones with the highest proliferation rate and possibly less differentiated cells. Increased expression of cell cycle drivers and reduced expression of some cell cycle inhibitors negatively correlate with GBM patient survival [56]. Interestingly, oxygen levels do not influence the expression of cell cycle genes in in vitro cultures, nor cell cycle distribution in cell cultures (Figure 3). Noteworthy, whereas lower oxygen concentration (1%) restricted cell division and we did not develop spheroid cell cultures, maintaining GBM cultures at 5% oxygen was permissive for cell proliferation.

Oxygenation level within tissues and in in vitro models is a subject of both debate and controversy [19,24]. Culturing cells as spheroids allows the generation of oxygen gradient, as shown previously by immunodetection of HIF1α and proteins dependent on HIF1α-driven transcription within GBM spheres cultured at 20% oxygen [57]. The enhanced expression of HIF1/2α inducible genes, like *ADM*, *ANGPTL4* or *CA9* in cells cultured at 5% oxygen indicates that this gradient is shifted in 5% oxygen cultures. Moreover, repeatable sphere dissociation in order to obtain single-cell suspension and subsequent regrowth of spheres creates a milieu of “cycling hypoxia”, shown to induce higher expression of HIF1/2α- driven genes when compared to a simple one-step reduction in oxygen level [10,58]. The experimental settings applied in this study better mimic the state within the tumour and simulate its oxygenation dynamics stemming both from the rapid growth of cellular masses and their vascularisation lagging behind. The set of hypoxia-sensitive genes up-regulated in 5% O_2_ vs. 20% O_2_ cultures is well represented in the tumour, and the expression of these genes is higher in GBM compared with normal brain and lower grade gliomas (Figure 6). As reported by Evans et al., measurement of oxygen tension in GBM, LGGs or normal brain showed that only GBM was hypooxygenated [11]. The most evident differences between transcriptomes of 5% and 20% O_2_ cultures are for canonical, HIF-1/2a activated genes coding for metabolic enzymes (Figure 5). Their expression is critical for cell survival when oxygen goes down, therefore this response must be robust and universal. However, our analysis specifies the genes consistently changing expression in low oxygen conditions that are not directly related to cell survival under a hypoxic insult, but potentially contribute to tumour evolution and therapy resistance. The exact role of increased expression in lower oxygen cultures is yet to be clarified.

Figure 2B,C show that most of the differentially expressed genes from EMT gene sets are canonical hypoxia-responsive genes, e.g., *VEGFA*, *COL1A2*, *SLC16A3*, and thus overlap with our findings presented in Figure 5 and Figure 6. Interestingly, the asymmetrical point distribution on volcano plots (Figure 2B,C) let us to hypothesise that other EMT genes may be up-regulated when cells are kept at 5% oxygen. Still, the lack of significance indicates that the response of investigated cell lines may be highly heterogeneous. Indeed, Joseph et al. demonstrated that some GBM cell lines responded to hypoxia with upregulation of EMT markers, whereas others did not [43]. It is worth noting that EMT in GBM differs from that occuring during organ development or epithelial cancer progression [59], and as such a subject of ongoing debate, also comprising the causative role of hypoxia [60]. Undoubtedly, exceptional intratumoural and intertumoural heterogeneity of GBM is one of the main obstacles to recognising all EMT aspects in this type of malignancy, also represented by our system.

Furthermore, lowering oxygen tension in tumour spheroid cultures offers protection against TMZ toxicity, and the increased expression of *MGMT* in 5% GBM cultures could be a likely reason for the observed resistance. Increased levels of MGMT protein in a hypoxic core of GBMs and its co-expression with CD133 were previously reported [40,61]. However, we did not find a correlation between the expression of *MGMT* and *CD133* in our dataset (not shown). Therefore, the exact mechanism of increased resistance of cells grown at 5% oxygen to TMZ remains to be elucidated. Our study and that of others suggest that cancer cells should be maintained under physiological oxygen conditions and is one of many voices convincing the scientific community that in vitro cancer research should be carried out under conditions as close to physiological as possible. The oxygen concentration is only one of the many parameters that ensure these conditions. Interactions between neoplastic cells and the extracellular matrix and other cells, e.g., the immune system are also important and should be a part of the more complex testing environment. These data contribute to understanding challenges in the design of an appropriate model for further studies on glioblastoma pathology and therapy.

The results described and discussed above strongly highlight the use of tumour-derived cultures in 5% oxygen for the investigation of therapy resistance mechanisms and the pursuit of new therapeutic targets and anti-GBM drugs. Moreover, lowered oxygen culture conditions could be applied to GBM, and other tumour-derived culture systems such as organoids or 3D scaffold cultures.

## 5. Conclusions

Here we report that lowering oxygen concentration from 20% to 5% during the establishment and maintenance of tumour-derived cell cultures allows for the long-term growth of GBM patient-derived 3D cell cultures. Transcriptome analysis of tumour and corresponding cell cultures kept at 5% or 20% oxygen showed increased cell cycle and DNA repair genes expression in cell cultures, and reduced expression of genes regulating inflammatory processes and neuronal function. We identified a group of genes significantly up-regulated in cell cultures maintained at 5% oxygen. The group of genes most significantly up-regulated at those conditions displayed a similar pattern of expression in 5% oxygen cell cultures and tumour samples but not in corresponding 20% oxygen cultures. Higher expression of these genes was also demonstrated in GBM samples in the TCGA dataset. 

Moreover, GBM cell cultures maintained at 5% oxygen were more resistant to TMZ. Our data demonstrate the advantages of culturing patient-derived GBM cells at 5% oxygen over cells maintained at 20% oxygen. The results provide a rationale for the application of such a setting for better modelling GBM cell behaviour in vitro.

## Figures and Tables

**Table 1:** The list of GBM patients, and obtained cell cultures with their characteristics (n.a., not available, e.g., no culture was established). Records highlighted in blue indicate samples analysed by RNA sequencing.

#	Sample ID	Patient Sex and Age	Cell Line	Culture Type
**1**	GCW01	M 62	None	n.a.
**2**	GCS6K1	F 66	5%	spheroid
**3**	GCSK2	F 45	5% and 20%	spheroid
**4**	GCS7K3	F 55	None	n.a.
**5**	GCW04	M 41	5% and 20%	spheroid
**6**	GCW07	M 62	5%	spheroid
**7**	GCW08	M 61	5%	spheroid
**8**	GCS19K6	F 51	5% and 20%	adherent
**9**	GCW10	M 48	5% and 20%	spheroid
**10**	GCW11	M 70	20%	spheroid
**11**	GCW12	F 74	None	n.a.
**12**	GCW14	F 57	None	n.a.
**13**	GCW16	F48	None	n.a.
**14**	GCW17	M 68	5% and 20%	adherent
**15**	GCW18	F 49	20%	adherent
**16**	GCW20	M 61	5% and 20%	spheroid
**17**	GCW21	M 68	5% and 20%	spheroid
**18**	GCW22	F 59	5% and 20%	spheroid
**19**	GCS29K7	F 55	5% and 20%	spheroid
**20**	GCW24	M 71	5% and 20%	spheroid
**21**	GCW25	M 57	5% and 20%	spheroid
**22**	GCW26	M 61	5% and 20%	spheroid
**23**	GCW27	F 66	5% and 20%	spheroid
**24**	GCW28	M 70	5% and 20%	spheroid
**26**	GCW29	F 63	5%	spheroid
**27**	GCW30	M 60	5% and 20%	spheroid
**28**	GCW31	M 63	5% and 20%	spheroid
**29**	GCW33	M 34	5% and 20%	adherent

Verhaak et al. classified GBM tumours into four transcriptional subtypes: mesenchymal, classical, proneural and neural [34]. In the current study, three out of ten tumours were defined as mesenchymal (GCW04, GCW21 and GCW27), however mesenchymal signature genes had their representation in most tumour specimens but not in the cell lines (Figure 1C). Cell lines cultured as spheroids represent a mixed proneural–classical subtype, except for GCW27 which has mostly mesenchymal characteristics.

## Data Availability

Sequence data have been deposited at the European Genome-phenome Archive (EGA), which is hosted by the EBI and the CRG, under accession number EGAS00001006267. Further information about EGA can be found on https://ega-archive.org “The European Genome-phenome Archive in 2021” (https://academic.oup.com/nar/advance-article/doi/10.1093/nar/gkab1059/6430505, accessed on 20 July 2022).

## Supplementary Materials

The following supporting information can be downloaded at: https://www.mdpi.com/article/10.3390/cancers14194852/s1, Table S1. KEGG pathways up- or down-regulated in 5% or 20% oxygen cell cultures vs tumour tissue; Table S2. KEGG pathways up-regulated in 5% vs. 20% oxygen for cell cultures only; Table S3. The list of genes expressed differentially between 5% and 20% oxygen cell cultures, with *p*-value < 1 × 10^−5^.
